# Biogenesis and homeostasis of mast cell lysosome related secretory granules

**DOI:** 10.3389/fcell.2025.1603999

**Published:** 2025-05-23

**Authors:** Ronit Sagi-Eisenberg

**Affiliations:** ^1^ Department of Cell and Developmental Biology, Gray Faculty of Medical and Health Sciences, Tel Aviv University, Tel Aviv, Israel; ^2^ Sagol School of Neuroscience, Tel Aviv University, Tel Aviv, Israel

**Keywords:** mast cells, lysosome related organelles (LRO), secretory granules, amphisomes, fusion, fission

## Abstract

Mast cells (MCs) are sentinel cells of the immune system that play important protective roles in innate host defenses but are also key effectors of allergic responses and chronic inflammatory diseases. Both physiological and pathophysiological responses of MCs are mediated by the release of inflammatory mediators, many of which are stored, preformed, in secretory granules (SGs), and released by regulated exocytosis in response to multiple stimuli. MC SGs belong to the family of lysosome related organelles (LROs), as indicated by their content of lysosomal hydrolases, lysosomal membrane proteins and acidic pH. The SGs derive from the Golgi and increase in size in a quantal manner by their fusion with additional SGs. They have access to external cargo, which they acquire by fusion with endosomes and contain LC3, which they acquire by fusion with amphisomes. This review discusses the underlying mechanisms of MC SG biogenesis and remodeling.

## 1 Introduction

Mast cells (MCs) are key regulatory cells of the immune system (Dahlin et al., 2022). Though best known for their critical role in allergy and anaphylaxis (Vitte et al., 2022), MCs also contribute to innate defense against infections and play significant roles in inflammatory conditions associated with autoimmunity, cancer, and neurodegenerative diseases (Theoharides et al., 2015; Segura-Villalobos et al., 2022; Hendriksen et al., 2017; Lin et al., 2023; Jiménez et al., 2021; St John and Abraham, 2013). MCs are particularly abundant at the interfaces between the external environment and the internal milieu, such as the skin, mucosa of the lungs, digestive tract, mouth, conjunctiva, and nose (Prussin and Metcalfe, 2006). Their presence in the brain has also been documented (Theoharides et al., 2024). In these locations, MCs are found in close proximity to blood vessels and sensory neurons. MCs originate from CD34^+^/CD117^+^ pluripotent progenitor cells in the bone marrow (Kirshenbaum et al., 1999). These progenitors migrate into peripheral tissues, where they mature and undergo terminal differentiation under the influence of local cytokines (Metcalfe et al., 1997; Prussin and Metcalfe, 2006). In connective tissues such as the skin, bone marrow–derived MCs progressively replace MCs that originated from extra-embryonic yolk sac and fetal liver (Chia et al., 2023).

MCs have been categorized into subsets based on their localization and protease expression profiles. In rodents, they are classified as mucosal MCs (MMCs) or connective tissue type MCs (CTMCs), whereas in humans, they are distinguished by their protease content: MC_TC_, which co-express tryptase and chymase among other proteases, and MC_T_, which express only tryptase (Reber et al., 2015). For both MC types, the transition from progenitor to mature MCs depends on activation of the c-KIT receptor upon binding of its ligand, stem cell factor (SCF) (Mekori et al., 1995). Additionally, both subsets express FcεRI, the high-affinity receptor for immunoglobulin E (IgE), which triggers MC activation upon allergen-induced crosslinking of cell-bound IgE (Dema et al., 2014; Nagata and Suzuki, 2022; Blank et al., 2021).

MC subsets differ not only in their protease expression profiles but also in their expression of Mrgprs, a family of G protein-coupled receptors selectively expressed in CTMCs or MC_TC_ (West and Bulfone-Paus, 2022; Ali, 2021; McNeil et al., 2015). These receptors enable IgE-independent activation in response to various ligands, previously termed MC basic secretagogues due to their positive charge (West and Bulfone-Paus, 2022; Ali, 2021; McNeil et al., 2015). These ligands include exogenous molecules such as toxins (e.g., the wasp venom peptide mastoparan), a wide range of FDA-approved drugs, such as vancomycin, and endogenous ligands such as neuropeptides (e.g., substance P) and antimicrobial peptides. Their activation of MCs can trigger pseudo-allergic reactions but also rapid innate immune and neorogenic responses (Subramanian et al., 2016). Notably, recent transcriptomic analyses of MCs from different tissues indicate that MC heterogeneity extends beyond their classification as MMCs or CTMCs, emphasizing the critical role of their microenvironment (Tauber et al., 2023; Akula et al., 2020). This aligns with evidence that MC activation is influenced by crosstalk with neighboring cells, including stromal cells, immune cells and neurons (Wang et al., 2024; Gri et al., 2012; Toyoshima and Okayama, 2022; Bao and Abraham, 2024). Interestingly, MCs are also present in the brain, where they interact with microglia, further highlighting their role in neuroimmune regulation (Huang et al., 2024; Hendriksen et al., 2017; Sandhu and Kulka, 2021).

Both the physiological immune responses of the MCs and their pathological functions in allergy and inflammation are primarily mediated by the release of inflammatory mediators, part of which, such as histamine, proteoglycans and proteases, are preformed and stored in secretory granules (SGs), that release their content immediately after activation by exocytosis. Others, such as prostaglandins, leukotrienes, cytokines and chemokines are synthesized *de novo* and released thereafter (Moon et al., 2014; Wernersson and Pejler, 2014; Blank et al., 2014; Gordon and Galli, 1990). Collectively, these mediators initiate early and late inflammatory responses.

## 2 The MC SGs

**FIGURE 1:**
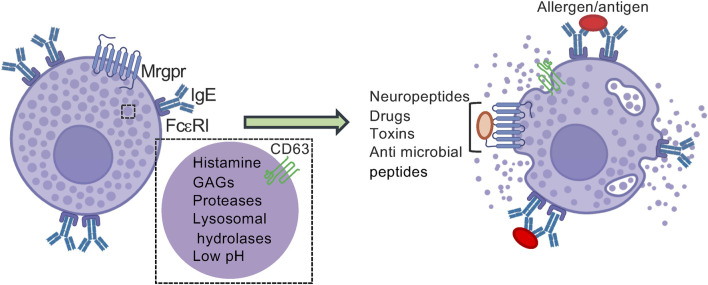
MC lysosome related SGs. MCs contain SGs which store preformed inflammatory mediators, including biogenic amines, such as histamine, serotonin and polyamines, proteoglycans, such as heparin and chondroitin sulfates, proteases such as tryptase, chymase and carboxypeptidase A3 and some cytokines, such as TNF-α. MC SGs also contain lysosomal enzymes, such as β-hexosaminidase and β-glucuronidase and lysosomal membrane proteins, such as CD63. In response to cell activation, for example, via the FcεRI, the high affinity receptor of IgE, via the binding of an allergen to cell bound IgE, or by ligands that bind to Mrgprs, G protein coupled receptors expressed in a subset of MCs, the SGs’ contents are released by exocytosis, a process referred to as degranulation. The inset is the enlargement of the boxed area. * “Created with BioRender.com”.

MC SGs belong to the family of lysosome related organelles (LROs), a specialized subset of SGs that exhibit lysosomal characteristics (Delevoye et al., 2019; Marks et al., 2013; Dell’Angelica et al., 2000; Luzio et al., 2014). The latter include secretory organelles of other immune cells, such as neutrophils, natural killer cells, and cytotoxic T lymphocytes, as well as melanocytes and osteoclasts, in which LROs play a role in pathogen killing, pigmentation and bone absorption (Delevoye et al., 2019; Marks et al., 2013; Dell'Angelica et al., 2000; Luzio et al., 2014). In neurons LROs are precursors of synaptic vesicles (Vukoja et al., 2018). Indeed, the MC SGs contain in addition to their inflammatory mediators, lysosomal enzymes (Schwartz and Austen, 1980) and lysosomal membrane proteins (LIMPs) (Suárez-Quian, 1987) and also contain an acidic luminal pH (Johnson et al., 1980) (Figure 1). The SGs also receive and exocytose in a regulated fashion endocytic cargo (Xu et al., 1998), recycle SG proteins (Bonifacino et al., 1989), and are regulated by endocytic recycling controlling synaptotagmins (Grimberg et al., 2003; Haberman et al., 2007). Based on electron microscopy (EM) analyses (Raposo et al., 1997), differential regulation by Soluble N-ethylmaleimide-sensitive-factor Attachment protein REceptors (SNAREs) (Adhikari et al., 2023; Xu et al., 2018; Lorentz et al., 2012), and fractionation data (Baram et al., 1999; Grimberg et al., 2003), MCs contain discrete types of SGs, which differ in their morphology (Raposo et al., 1997) and content composition (Puri and Roche, 2008). Specifically, based on their transmission EM features, the SGs were classified into three types: Type I granules, which contain intraluminal vesicles (ILVs), reminiscent of multivesicular bodies (MVBs), and become accessible to external cargo after a 20-min lag, Type II granules which display a serotonin-rich electron-dense core surrounded by ILVs and become accessible to endocytic cargo at a later stage, and Type III granules, which are electron-dense and lack ILVs (Raposo et al., 1997). Both Type I and Type II granules also contain MHC class II molecules, mannose-6-phosphate receptors, and lysosomal membrane proteins (Lamp1 and Lamp2), which localize to the small intraluminal vesicles (Raposo et al., 1997).

## 3 The relationship between lysosomes and the lysosome related SGs in MCs

**FIGURE 2:**
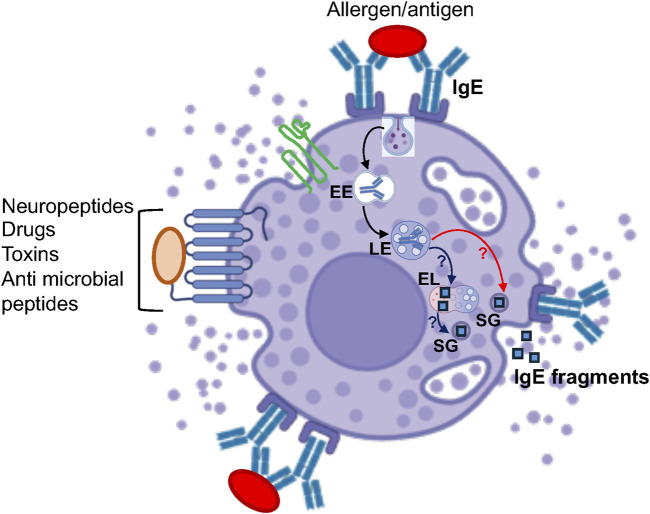
Model of the potential crosstalk between MC SGs and the endolysosomal system. According to this model, after internalization, external cargo such as IgE travels from early endosomes (EE) to late endosomes (LE) and is degraded in endolysosomes (EL). This degraded cargo may then be delivered to the SGs, possibly via SG-EL fusion (blue arrows), followed by its release during exocytosis. Alternatively, internalized cargo might be directly delivered from late endosomes to the SGs, which are proteolytically active, where it is degraded before being released. * “Created with BioRender.com”.

Previous studies have demonstrated the presence of acid phosphatase in two populations of granules, one which comprised most granules, also exocytosed, while the other smaller one, was retained in triggered cells (Jamur and Vugman, 1990). Similarly, the enzyme Dipeptidyl aminopeptidase II (DAP II) was found to reside in few granules that reside near the nucleus and are retained in MCs that are triggered to degranulate (Sannes and Spicer, 1979). By cell fractionation, we have demonstrated the existence of two types of β-hexosaminidase containing fractions, one that also contains histamine, while the other is histamine-free (Baram et al., 1999). However, while these studies may imply the existence of lysosomes that are distinct from the SGs, fragments of IgE, that was bound to the IgE receptor, were shown to be released during exocytosis (Xu et al., 1998), suggesting that endocytosed IgE is degraded and delivered to the SGs or degraded in the SGs. Therefore, the precise relationship between degradative endolysosomes and the lysosomal related SGs is still poorly resolved (Figure 2).

## 4 The crosstalk between MC SGs and the autophagic system

Further complexity in the mechanisms underlying MC SG biogenesis is highlighted by the presence of LC3 within SGs, where it colocalizes with the tetraspanin protein CD63 and is released during exocytosis (Nakano and Ushio, 2011). This observation indicates the existence of crosstalk between the autophagic system, which is constitutively active in MCs (Nakano and Ushio, 2011), and the SGs. Indeed, we have recently demonstrated dynamic interactions between SGs and the autophagic system, as evidenced by the targeting of MRGPRX2, the human member of the Mrgpr receptor family, to LC3-positive SGs following its substance P-induced internalization. Moreover, the number of LC3-positive SGs increases in response to receptor internalization (Lazki-Hagenbach et al., 2022). While the precise nature of these interactions remains to be fully elucidated, at least one mechanism involves SG fusion with amphisomes, as discussed below.

## 5 The biogenesis of MC SGs

### 5.1 MC SGs undergo dynamic remodelling

**FIGURE 3:**
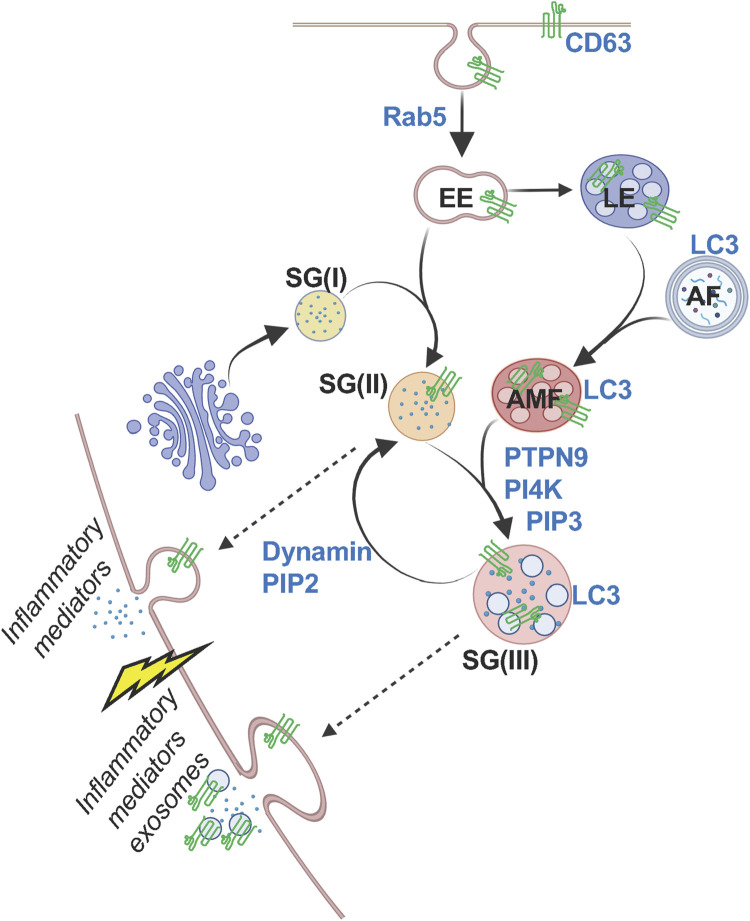
Model of the biogenesis of MC SGs. According to this model, Golgi derived SGs [SG(I)] incorporate endocytic cargo, including CD63, which internalized from the plasma membrane by a Rab5-regulated mechanism, by fusion with early endosomes (EE). The fused SGs [SG (II)] can further fuse by a mechanism dependent on the protein tyrosine phosphatase PTPN9, CD63 and phosphatidylinositol-4-kinase (PI4K), with amphisomes (AMFs), which form by the fusion of late endosomes (LE) with autophagosomes (AFs), forming large and LC3-positive SGs [SGIII)] that also contain intraluminal vesicles. Both SG (II) and SG (III) are exocytosis competent. However, in response to an external trigger, SG (II) release prestored inflammatory mediators, while SG (III) additionally release CD63-positive exosomes. SG (III) can revert to SG (II) by undergoing dynamin-mediated fission. Dynamic cycling of the SGs between fusion and fission events is regulated by phosphoinositides interconversion between PIP3 and PIP2. * “Created with BioRender.com”.

Early EM analyses of MCs (Combs, 1971), further supported by our analyses of the route of trafficking of Neuropeptide Y (NPY)-mRFP to the SGs (Azouz et al., 2012), demonstrate that the SGs derive from the Golgi, implying dynamic interactions between Golgi derived granules and the endocytic and autophagic systems. Subsequent analyses of EM images implicated fusion between SGs as a key post-Golgi mechanism for generating mature SGs (Hammel et al., 2010). In agreement with this model, we found that expression of a constitutively active mutant of the small GTPase Rab5 leads to SG enlargement that is linked with a reduction in SG number (Azouz et al., 2014). Conversely, Rab5 knockdown reduces the SG size while increasing the SG number (Azouz et al., 2014). These findings implicate Rab5 in the regulation of SG fusion. Furthermore, we have demonstrated that Rab5 also facilitates SG fusion with endosomes, allowing the incorporation of CD63 into the SGs (Azouz et al., 2014). This reinforces the idea that fusion events between Golgi-derived SGs contribute to their enlargement and the incorporation of both external and endogenous endocytic cargo. Further analysis of Rab5-mediated SG fusion has unveiled key steps in this process, clarifying several previously enigmatic observations (Omari et al., 2024). Specifically, we found that homotypic SG fusion is a multistep process that requires CD63 and depends on Rab5-regulated CD63 internalization. Additionally, SG enlargement necessitates fusion with amphisomes, hybrid organelles formed by the fusion of late endosomes with autophagosomes, highlighting further the close interplay between the SGs, late endosomes and autophagosomes (Figure 3). This mechanism explains the colocalization of LC3 with CD63 at SGs, as well as prior observations, which documented the release of mitochondrial fragments during MC exocytosis (Zhang et al., 2012). In addition to its dependence on CD63, SG fusion with amphisomes is regulated by phosphatidylinositol (PI) 3- and 4-kinases, as well as the protein tyrosine phosphatase PTPN9 (PTP-MEG2), which has previously been implicated in granule fusion (Omari et al., 2024). SG fusion is also associated with an enrichment of PI(3)P, PI(4)P, and PI(3,4,5)P_3_ in the SG membrane, of which the latter may be required for the activation of PTPN9, as its interaction with phosphoinositides is essential for relieving its autoinhibition (Kruger et al., 2002). Strikingly, we found that fusion with amphisomes not only enlarges SGs, allowing them to store greater amounts of secretory cargo that is “ready to go” during degranulation, but also endows SGs with the ability to release exosomes (Omari et al., 2024) (Figure 3).

Unexpectedly, we discovered that SGs also undergo fission through a dynamin-mediated mechanism, which is triggered by a switch from SG PI(3,4,5)P_3_ to PI(4,5)P_2_ (Omari et al., 2024) (Figure 3). Taken together, these findings suggest that MCs may harbor SGs of varying sizes and contents, depending on their fusion and fission events. Some smaller SGs may only release soluble mediators, potentially through kiss-and-run exocytosis, while larger SGs may release both soluble mediators and exosomes, likely via compound exocytosis, which requires a more stable fusion pore opening (Flašker et al., 2013). The relative abundance of distinct SG subtypes is likely regulated by factors such as the activation state of relevant lipid kinases and phosphatases, which determine the SG phosphoinositide composition, as well as the extent of internalization of CD63 and the cellular levels of amphisomes (Figure 3).

### 5.2 The role of protein recycling

#### 5.2.1 The role of LYST

Chediak-Higashi syndrome (CHS) is an autosomal recessive disorder caused by mutations in the Lysosomal Trafficking Regulator (LYST) gene, which lead to a broad range of clinical manifestations associated with the enlargement of lysosomes and LROs, including MC SGs (Turner et al., 2024; Shiflett et al., 2002; Kiyoi et al., 2019). Mechanistically, LYST has been implicated in regulating lysosome/LRO size by promoting fusion or inhibiting fission. It has also been linked to controlling the movement of lysosomes and LROs (Turner et al., 2024; Serra-Vinardell et al., 2023). However, the precise mechanism underlying LYST’s function remains unresolved. Interestingly, unlike the functional impact of LYST mutations on the lytic granules of cytotoxic T lymphocytes or NK cells, which results in reduced cytotoxicity (Turner et al., 2024), analyses of skin and peritoneal MCs (i.e., CTMCs) and bone marrow-derived MCs (i.e., BMMCs) from homozygous Beige mice carrying a mutation in LYST revealed enlarged SGs, which despite this enlargement, preserved their exocytosis competence (Kiyoi et al., 2019). This observation is consistent with our findings showing that the SG size has no impact on their exocytosis competence (Omari et al., 2024).

#### 5.2.2 Regulation by synaptotagmins

A role for endocytic recycling in the biogenesis of MC SGs is suggested by the influence of certain members of the synaptotagmin (Syt) family of proteins on their biogenesis. Seventeen members of this family have been identified based on their common structural features, which include a short lumenal/extracellular domain, a transmembrane or membrane association domain (for Syt16 and Syt17), and two cytoplasmic C2 calcium-binding domains (Wolfes and Dean, 2020). Syts have been implicated in regulating protein trafficking along both exocytic and endocytic routes (Wolfes and Dean, 2020). In MCs, knockdown of Syt III, which interfered with the transport of internalized transferrin to the endocytic recycling compartment (ERC), induced a Chediak-Higashi-like phenotype, characterized by a significant increase in the number of giant SGs (Grimberg et al., 2003). Knockdown of Syt IX, which disrupted the recycling of transferrin (Tfn) from the ERC to the plasma membrane (Haberman et al., 2003), led to the mistargeting of TGN38 to the SGs (Haberman et al., 2007). These findings support a model in which endocytic recycling plays a role in segregating endosomal cargo, preventing its accumulation in SGs. Spillover of cargo from the ERC to late endosomes may result in mistargeting of TGN cargo to the SGs, possibly via the formation of amphisomes.

### 5.3 The role of Hermansky-Pudlak Syndrome genes

Hermansky-Pudlak Syndrome is a group of autosomal recessive disorders characterized by oculocutaneous albinism, bleeding disorders, innate immune deficiency and pulmonary fibrosis, all of which are associated with abnormalities in LRO biogenesis in melanocytes, platelets, neutrophils, natural killer cells and cytotoxic T lymphocytes (Bowman et al., 2019; Wei, 2006; Banushi and Simpson, 2022; Starcevic et al., 2002). The disease is caused by genetic defects in 11 different genes that encode subunits of protein complexes involved in the biogenesis of LROs (Banushi and Simpson, 2022). These include subunits of the Biogenesis of Lysosome-related Organelles Complexes (BLOC)-1, -2, and -3, as well as the β subunit of the adaptor complex AP-3 (Dell’Angelica, 2009; Banushi and Simpson, 2022). Analysis of dermal MCs and an HPS-1-derived MC culture revealed abnormalities in SG morphology and an increase in activation markers (Kirshenbaum et al., 2016). These findings suggest a role for HPS-1, a subunit of BLOC-3, in the biogenesis of MC SGs, although the precise mechanism remains to be further investigated. In a separate study, the role of the AP-3 complex was examined. This complex is part of the AP-1 to AP-5 family of adaptor protein complexes, which mediate the transport of distinct types of vesicles (Dacks and Robinson, 2017; Begley et al., 2024). The complexes are structurally related consisting of α/γ/δ/ε/ζ, β1-5, μ1-5, and σ1-5 subunits (Begley et al., 2024). Among these adaptor complexes, the AP-3 complex has been implicated in regulating transport from endosomes to lysosomes and LROs (Begley et al., 2024). shRNA-mediated knockdown of the δ subunit of AP-3 in RBL-2H3, a mast cell line widely used as a model for MC exocytosis (Falcone et al., 2018), destabilized the complex, leading to its depletion (da Silva et al., 2017). Morphometric evaluation of the SGs by EM revealed an increase in SG size (da Silva et al., 2017), like the phenotype observed with Syt III depletion (Grimberg et al., 2003). AP-3 may thus play a role in the cellular transport of Syt III and potentially other membrane proteins to the SGs.

Two other proteins linked to HPS are the Rab GTPases Rab32 and Rab38, for which the BLOC-3 complex displays GEF activity. Indeed, both Rabs have been implicated in the biogenesis of LROs in several cell types (Bultema and Di Pietro, 2013). In MCs, expression of a constitutively active mutant of Rab38, but not Rab32, selectively inhibited IgE-mediated degranulation, while it had no effect on MRGPRX2-or calcium ionophore and phorbol ester-induced release (Azouz et al., 2012). However, expression of neither Rab32 nor Rab38 affected the SG size (Azouz et al., 2012; Lazki-Hagenbach et al., 2024).

## 6 The regulation of MC SG transport

Similar to lysosomes and LROs in other cell types, the SGs in MCs move bidirectionally (Smith et al., 2003). Additionally, similar to other LROs, the anterograde transport of MC SGs is regulated by Rab27. However, in cytotoxic T lymphocytes, the anterograde movement of lytic granules to the immune synapse is mediated by Rab27a, which recruits kinesin-1 through its effector synaptotagmin-like protein 3 (Slp3) (Kurowska et al., 2012). In contrast, in MCs, kinesin-1 is recruited to SGs via the Rab27b–Slp3 complex (Munoz et al., 2016). Furthermore, in MCs this recruitment is dependent on PI3K activity and accordingly occurs only in activated cells (Munoz et al., 2016). Another regulator of kinesin-1-mediated translocation of the SGs to the plasma membrane in activated cells is the large GTPase Rab44 (Longé et al., 2022). The precise relationship between these two mechanisms of SG transport remains unknown. Finally, and most intriguingly, SG trafficking to the plasma membrane in activated cells was shown to require the association of inflammasome components with SGs (Mencarelli et al., 2024). Moreover, this mechanism also involves the motor protein dynein (Mencarelli et al., 2024), which has previously been implicated in the retrograde transport of MC SGs (Efergan et al., 2016). Notably, MC SGs differ from other LROs in their mechanism of retrograde transport. While Rab7 and Rab36 mediate the recruitment of the RILP–dynein complex to other LROs (Daniele et al., 2011; Matsui et al., 2012), in MCs this role is fulfilled by Rab12 (Efergan et al., 2016). Intriguingly, Rab12 also stimulates SG translocation to cell tips in activated cells (Efergan et al., 2016). How Rab12 and dynein can simultaneously promote perinuclear accumulation of a subset of SGs while driving translocation of another subset to the cell surface remains unknown. It is noteworthy that this type of dual regulation is not without precedent: Rab7 has been shown to drive lysosome movement in either direction by binding different effectors, depending on the cellular concentration of cholesterol (Rocha et al., 2009). Whether Rab12 controls the anterograde transport of SGs through effectors other than RILP, and how Rab12, Rab27b, Rab44 and inflammasome-regulated transport are functionally related, remain open questions. It is also worth noting that Rab12 is one of the physiological substrates of the leucine-rich repeat kinase 2 (LRRK2), a kinase whose hyperactivation is linked to Parkinson’s and Crohn’s diseases (Hui et al., 2018). However, whether phosphorylation of Rab12 plays a role in regulating MC functions is currently unknown.

## 7 SG homeostasis

### 7.1 The role of serglycin

Proteoglycans containing glycosaminoglycan (GAG) side chains of either heparin or chondroitin sulfate are central components of MC SGs, with serglycin serving as the core protein. Serglycin features an extended Ser-Gly repeat region, in which each Ser-Gly unit provides a potential GAG attachment site (Rönnberg et al., 2012). Strikingly, knockout of serglycin impairs the storage of proteases and histamine leading to disorganized SGs (Abrink et al., 2004), highlighting serglycin’s key role in the retention of proteases and histamine within SGs. Interactions between serglycin and SG proteins prevent premature degradation and regulate the kinetics of their diffusion into the extracellular milieu following triggered exocytosis. Cargo with a high affinity for serglycin is retained near the SG, while low-affinity cargo, such as β-hexosaminidase, diffuses away into the circulation. Based on our findings on SG fission, we hypothesize that serglycin may also contribute to the well-documented heterogeneity of SGs. This could occur through the unequal distribution of granule contents between budding SGs, driven by differential binding affinities of cargo molecules to serglycin (Rönnberg et al., 2012). Interestingly, deficiency of serglycin-dependent proteases reduces the amount of heparin, replicating the phenotype of serglycin deficiency by causing a major distortion in SG integrity, presumably due to a disruption in the SG’s electric charge balance (Grujic et al., 2013).

### 7.2 The role of acidic pH

Significant morphological changes were also observed in MCs treated with bafilomycin A1, an inhibitor of the vacuolar-type ATPase proton pump (Pejler et al., 2017). The granules became swollen and acquired a vacuole-like morphology (Pejler et al., 2017). Bafilomycin A1 also had selective effects on SG cargo, altering the processing of pro-carboxypeptidase A3, reducing the level of SG-stored histamine, and enhancing the autoproteolysis of tryptase (Pejler et al., 2017). In contrast, the storage of β-hexosaminidase was unaffected. Therefore, a low SG pH is essential for maintaining the homeostasis of MC SGs (Pejler et al., 2017).

## 8 The impact of aging

MC numbers increase in aged tissue and changes have been recorded in their responsiveness and ability to degranulate (Chatterjee and Gashev, 2012; Pilkington et al., 2019). To gain insights into the autonomous changes that may occur in MCs during aging, we recently established a novel model of inducible senescence in MCs as a paradigm of aging (Kleeblatt et al., 2024). This model is based on the inducible upregulation of the cell cycle inhibitor p16INK4A, which we have also shown to be upregulated in human skin derived from elderly donors and in peritoneal MCs derived from old mice (Kleeblatt et al., 2024). Analyses of in vitro-differentiated MCs derived from the bone marrow of these transgenic mice revealed significant morphological and functional differences in the SGs of senescent MCs. These differences were reflected in a significant increase in large SGs containing intraluminal vesicles (ILVs), which was associated with a shift towards the regulated release of smaller, CD63-enriched extracellular vesicles (EVs), reminiscent of the functional changes observed following SG fusion with amphisomes (Omari et al., 2024). Interestingly, this increase in the release of small CD63-positive EVs was also associated with an increase in proteoglycan exteriorisation, while the ability to release β-hexosaminidase decreased during prolonged senescence (Kleeblatt et al., 2024).

## 9 Conclusion and perspectives

LROs were traditionally defined as a subtype of SGs that exhibit lysosomal features. However, this group of organelles encompasses a variety of structures that appear to differ in their mechanisms of biogenesis. Furthermore, the boundaries between conventional SGs, LROs and lysosomes have become more fluid, as accumulating data demonstrate the involvement of both the endocytic and autophagic systems in the biogenesis of endocrine and exocrine SGs (Patel et al., 2013; Bel et al., 2017; Morishita et al., 2020; Li et al., 2022). Furthermore, classical lysosomes can also undergo exocytosis (Trojani et al., 2024). Therefore, while LROs may not necessarily share common mechanisms for their biogenesis, some of these mechanisms might be shared with conventional SGs, classical lysosomes, or autolysosomes. Deciphering the molecular details of the interactions between MC SGs and the endolysosomal and autophagic systems could contribute to our understanding of these processes in other cells. In this review, we primarily focused on factors shown to play a role in the biogenesis of MC SGs. However, many questions remain unanswered. For example, how are proteins targeted to the SGs? Do specific sorting signals direct SG cargo to SGs rather than lysosomes, or do lipid phase separations play a role in cargo targeting? What is the precise role of CD63, PI4K, and PTPN9 in mediating SG fusion? What is the exact role of Lyst or synaptotagmins? What is the precise mechanism of SG fission, and which lipid kinases and phosphatases control the switch between fusion and fission? What is the relationship between SGs and degradative endolysosomes or autolysosomes? How do Rab GTPases that affect SG size or exocytosis execute their regulatory functions? What is the precise mechanism of SG recapture and regranulation? Finally, what is the precise mechanism of SG exocytosis? a process that remains incompletely understood. Future studies, leveraging novel technologies and tools, will need to address these fundamental questions to better understand the functions of MCs in health and disease.
